# Supplementary material for: Ethnobotanical Documentation of the Uses of Wild and Cultivated Plants in the Ansanto Valley (Avellino Province, Southern Italy)
Source: Plants (Basel). 2023 Oct 26;12(21):3690. doi: 10.3390/plants12213690 (PMC10649993; doi:10.3390/plants12213690)
Supplement: Supplementary file 1 [file plants-12-03690-s001.zip › plants-2650422-supplementary.pdf]

Table S1. Traditionally used plants in the areas of Ansanto Valley (U.R.: Use Reports; Cos: cosmetic uses; Cul: culinary uses; Med: medicinal uses; Vet: veterinary uses).

**Name (Family)**	**Vernacular name**	**Parts used**	**Preparation**	**Administration**	**Use**	**U.R.**
Achillea millefolium L. (Asteraceae) AV-001	Achiella, Evera d'Autiello	Flowers	Decoction	Oral	**Med:** ulcers	**1**
			Infusion		**Med**: varicose veins	**1**
					**Med**: gastritis	**1**
		Whole plant	Decoction		**Vet:** against the intestinal parasites of calves	**1**
Agrimonia eupatoria L. (Rosaceae) AV-002	Erba delle scottature, evera de lu cuotto	Stem, Leaves, Flowers	The dried and finely chopped plant is toasted, then mixed with (or without) lard and applied on the burns	Topical	**Med**: burns	**6**
Allium sativum L. (Amaryllidaceae) AV-003	Agghie, Aglio	Bulbs	Raw	Oral	**Med**: intestinal disinfectant, against intestinal worms	**5**
					**Med**: blood pressure	**4**
					**Med**: antimicrobial	**2**
					**Med**: tonic	**1**
			Raw bulbs crushed in a mortar with rue leaves and then filtered		**Med:** stomach and belly ache	**5**
			Raw	Topical	**Med**: insect bites	**3**
			Raw bulbs crushed with olive oil		**Med**: to remove calluses	**1**
			Raw		**Dom**: insects repellent	**2**
					**Dom:** bulbs carried in a pocket to keep snakes away	**1**
Aloe arborescens Mill. (Asphodelaceae) AV-004	Aloe	Mucilaginous tissue	Raw	Topical	**Med**: burns	**2**
					**Med**: skin lenitive	**1**
					**Med**: insect bites	**1**
					**Med**: to heal sores in the mouth	**1**
					**Cos**: skin tonic	**1**
Apium graveolens L. (Apiaceae) AV-005	Sedano selvatico	Stem, leaves			**Cul:** as vegetable, aromatizer	**2**
Artemisia absinthium L. (Asteraceae) AV-006	Assenzio, Nascienzo	Leaves	Decoction	Oral	**Med**: intestinal warms	**2**
		Leaves, Flowers	Infusion		**Med**: febrifuge, antiseptic	**2**
		Flowers	Macerate (in wine)		**Med**: aperitif	**1**
		Leaves	Liquor		**Med**: digestive	**1**
Arundo donax L. (Poaceae) AV-007	Canne	Stems			**Dom:** baskets	**4**
Asparagus acutifolius L. (Asparagaceae) AV-008	Asparagi, Sparici	Turions	Cooking water	Oral	**Med**: diuretic	**2**

			Eaten cooked		**Med**: iron supplements	**1**
					**Cul**: as vegetable	**4**
Avena sativa L. (Poaceae) AV-009	Avena	Fruits	Boiled with barley, the water is drunk warm	Oral	**Med**: lower cholesterol	**1**
			Boiled, the water is drunk warm		**Med**: sedative	**1**
		Stems			**Dom**: straw sculptures	**1**
					**Dom:** drinking straws	**1**
Bellis perennis L. (Asteraceae) AV-010	Margherita, Margheritine, Margarita	Leaves	The raw leaves are crushed and applied topically	Topical	**Med**: pimples healing	**3**
		Leaves, Flowers	Decoction	Oral	**Med**: mouth and throat inflammations	**2**
		Whole plant			**Med**: to treat acne	**1**
		Flowers	Infusion		**Med**: refreshing	**1**
			Infusion	Topical	**Cosm**: to make eyes bright	**1**
		Whole plant	Decoction in milk		**Cos:** facial cleansing	**1**
Beta vulgaris L. subsp. *maritima* (L.) Arcang. (Amaranthaceae) AV-011	Bietola, Ieta	Leaves	Decoction	Oral	**Med**: rheumatism	**1**
					**Med**: blood circulation	**1**
					**Med**: diuretic	**1**
					**Cul**: as vegetable	**12**
Borago officinalis L. (Boraginaceae) AV-012	Borragine, Vorraine, Vorraena, Borraine	Leaves	Decoction	Oral	**Med**: rheumatism	**3**
					**Med**: blood circulation	**1**
					**Med**: cough	**1**
					**Cos:** skin purification	**1**
			Raw in the feed		**Vet**: galactagogue for cows	**2**
					**Cul**: as vegetable	**17**
Brassica oleracea L. (Brassicaceae) AV-013	Cavolo selvatico	Leaves	Crushed ina mortar	Topical	**Med**: burns	**1**
		Leaves, flowers			**Cul**: as vegetable ("Pizza gialla", "Pizza e minestra", "Pizza sciatizza"	**2**
Brassica cretica Lam. [B. oleracea L. var. botrytis L.] (Brassicaceae) AV-014	Cavolfiore	Leaves	Crushed and applied to the knee	Topical	**Med**: synovitis	**1**
Brassica rapa L. (Brassicaceae) AV-015					**Cul**: as vegetable in pizzas	**1**
Calendula officinalis L. (Asteraceae) AV-016	Calendula	Flowers	Infusion	Oral	**Med**: sedative, headache	**1**
		Stems, Leaves, Flowers	Crushed in a mortar and topically applied	Topical	**Med**: burns	**1**

		Leaves			**Cul**: as vegetable	**1**
		Flowers	Petals crushed in a mortar and used as saffron		**Cul**: as spice	**1**
Camellia sinensis (L.) Kuntze (Theaceae)	Tè nero	Leaves	Boiled in the water		**Dom:** fabric dye	**1**
Cannabis sativa L. (Cannabaceae) AV-017	Canapa	Stems	The fibers kneaded with egg and sugar to block the joints in case of sprains	Topical	**Med**: sprains	**5**
		Flowers	Fresh flowers chewed		**Med**: toothaches	**2**
		Stems	Stems are soaked in water streams, then dried, broken and scutched to separate the desired fibers from hemp's woody core		**Dom:** for making ropes, fabrics, tow, sheets, seamless stockings	**5**
Capparis spinosa L. (Capparaceae) AV-018	Capperi	Tips of the branches, immature fruits.		Brine	**Cul**: flavoring	**1**
		Flower buds		Salted	**Cul**: flavoring	**3**
Capsicum annuum L. [C. longum L.] (Solanaceae) AV-019	Peperoncino	Fruits, seeds	Raw	Oral	**Med**: coronary arteries cleansing	**1**
					**Cul**: condiment	**13**
Castanea sativa Mill. (Fagaceae) AV-020	Castagne	Seeds	Decoction with barley and a not identified "erba dei muretti"	Oral	**Med**: to calm cough	**1**
Centaurium erythraea Rafn. (Gentianaceae) AV-021	Centaurea	Flowered aerial parts	Decoction	Oral	**Med**: febrifuge	**3**
Chelidonium majus L. (Papaveraceae) AV-022	Celidonia	Latex	Raw	Topical	**Med**: to remove thorns under the skin	**1**
					**Med**: warts	**4**
Chenopodium album L. (Amaranthaceae) AV-023	Farinole	Leaves	Infusion	Oral	**Med:** diuretic	**1**
		Leaves			**Cul**: as vegetable	**3**
Chenopodium bonus-enricus L. (Amaranthaceae) AV-024	Orapi	Leaves			**Cul**: as vegetable	**1**
Cichorium intybus L. (Asteraceae) AV-025	Cicoria, Cecoria	Leaves	Decoction	Oral	**Med**: liver depurative	**8**
					**Med**: diuretic	**4**
					**Med**: digestive	**2**
					**Med**: cure the after-effects of bronchitis	**2**
					**Med**: viral infections	**2**
					**Med**: invigorating	**2**
					**Med**: diabetes	**1**
					**Med**: lower cholesterol	**2**
					**Med**: blood pressure	**2**
					**Med:** purify blood after hangover	**1**

					**Cul**: as vegetable in soups and pizzas	**22**
		Roots	Dried roots are ground and used as coffee powder		**Cul:** coffee	**3**
Citrus x *limon* (L.) Osbeck (Rutaceae) AV-026	Limone	Fruit	Juice in hot water	Oral	**Med**: dysentery	**1**
			Juice added to coffee (no sugar)		**Med**: headache	**1**
			Lemon and pomegranate juice		**Med**: refreshing	**1**
			Liquor		**Med**: digestive	**1**
			Juice with salt	Topical	**Med**: epistaxis	**1**
			Juice		**Dom:** invisible ink	**1**
			Juice with water and sand		**Dom:** clean copper pots	**2**
Clematis vitalba L. (Ranunculaceae) AV-027	Vetaglie	Young shoots			**Cul**: as vegetable	**2**
Clinopodium nepeta (L.) Kuntze subsp. *nepeta* (Lamiaceae) AV-028	Nepeta	Leaves			**Cul**: flavoring	**21**
Coffea arabica L.; *C. canephora* Pierre ex A.Froehner (Rubiaceae)	Caffè	Seeds	See *Citrus* x *limon*	Oral	**Med**: headache	**1**
Cucumis sativus L. (Cucurbitaceae)	Cetriolo	Fruits	Fresh slices on the face	Topical	**Cos:** beauty masks, skin tonic	**1**
Cynara cardunculus L. subsp. *cardunculus* (Asteraceae) AV-029	Cardo	Leaves, stems		Oral	**Vet**: goat feed	**3**
Cynara cardunculus L. subsp. *scolymus* (L.) Hegi (Asteraceae)	Carciofo	Flower buds		Oral	**Med**: digestive	**1**
					**Med**: liver depurative	**1**
Ecballium elaterium (L.) A.Rich (Cucurbitaceae) AV-030	Cocomero asinino, Scattapiatti, Cucuruzzuli	Fruit	A cotton swab is soaked in the fruit juice and inhaled through the nose	Inhalation	**Med**: sinusitis, cold	**3**
					**Med**: conjunctivitis	**1**
					**Med**: otitis	**1**
					**Med**: sniffed to sleep	**1**
Cynodon dactylon L. (Poaceae) AV-031	Gramigna, Ramegna	Rhizome	Decoction (with bay laurel leaves, mauve leaves and chamomile flowers)	Oral	**Med**: bellyache	**2**
			Decoction		**Med**: diuretic, cystitis, gout	**5**
					**Med**: back pain, toothache	**1**
					**Med**: blood depurative	**1**
					**Med**: rheumatism	**1**
				Topical	**Med**: eczema	**1**
				In the horses and rabbits feed	**Vet**: make the coat shinier, increase the immune system	**3**

			Boiled		**Cul**: base for jellies, alcohol and beer	**1**
Diplotaxis tenuifolia (L.) DC. (Brassicaceae) AV-032	Rucola	Leaves			**Cul**: salads, pasta condiment	**8**
Eriobotrya japonica (Thunb.) Lindl. (Rosaceae) AV-033	Nespolo	Flowers	Decoction	Oral	**Med**: flu, cold	**2**
Ficus carica L. (Moraceae) AV-034	Fico	Latex	Raw	Topical	**Med:** insect bites	**5**
					**Med**: warts, skin fibroids	**2**
		Fruits			**Cul**: jam	**1**
					**Cul**: grappa aromatizer	**1**
Foeniculum vulgare Mill. (Apiaceae) AV-035	Finocchietto, Finocchio selvatico, Finocchiastro	Fruits	Decoction	Oral	**Med:** bellyache	**5**
					**Med**: toothache	**2**
					**Med:** digestive	**3**
					**Med**: diuretic	**1**
					**Med**: flu	**1**
					**Med**: cold	**2**
					**Med:** sore throat	**1**
					**Med**: sedative	**1**
		Fruits			**Cul**: liqueur	**3**
					**Cul:** aromatizer in taralli and salami	**17**
		Leaves			**Cul**: as vegetable	**1**
Genista tinctoria L. (Fabaceae) AV-036	Ginestra, Inesta, Ianesta	Flowers	Raw	Topical	**Med**: nosebleed	**1**
		Stems			**Dom:** brooms	**2**
					**Dom**: to make clothes	**3**
Glaucium flavum Crantz (Papaveraceae) AV-037	Papagno giallo	Whole plant	Decoction	Oral	**Med**: insomnia	**1**
Hedera helix L. (Araliaceae) AV-038	Edera	Leaves	Infusion	Oral	**Med**: bronchitis	**1**
			Maceration in olive oil	Topical	**Med**: burns	**1**
			Leaves put in warm tub water before bathing at least twice a week		**Med**: stretch marks and cellulite	**2**
			Decoction (the filtered water is used to rinse the hair after shampooing)		**Cos:** darken hair	**1**
			Decoction (the garments are soaked in filtered water)		**Dom:** prevent wool felting	**1**
Hedysarum coronarium L. (Fabaceae) AV-039	Sulla	Leaf rachis	Chewed raw	Oral	**Med**: for refreshment during field work	**1**
Hordeum vulgare L. (Poaceae) AV-040	Orzo	Fruits	Boiled with oats, the water is drunk warm	Oral	**Med**: cough	**3**
			The caryopsis are roasted and ground, then boiled and filtered, sometimes oats is added		**Med**: sore throat	**4**
			Decoction		**Med**: cold	**1**

			Boiled with oats		**Med**: calming for children	**2**
Hypericum perforatum L. (Hypericaceae) AV-041	Iperico, Evera de riumo (erba del rumine)	Petals	Maceration in olive oil	Topical	**Med**: burns	**1**
		Whole plant	Decoction	Oral	**Vet**: free the cows' rumen	**2**
Junglans regia L. (Juglandaceae) AV-042	Noce	Fruits	Liquor	Oral	**Med**: stomach ache, digestive	**10**
			Liquor	Oral	**Cul**: liqueur	**5**
			Minced walnut green husk	Topical	**Cos:** to dye hair	**2**
		Wood			**Dom:** barrels for transporting water	**3**
Lactuca sativa L. (Asteraceae) AV-043	Lattuga	Leaves	Decoction with *Matricaria chamomilla*	Topical	**Med**: conjunctivitis	**1**
		Leaves	Decoction		**Med**: mouthwash	**1**
		Leaves	Boiled leaves applied on tooth		**Med**: toothache	**3**
Lactuca sativa subsp. *serriola* (L.) Galasso, Banfi, Bartolucci & Ardenghi (Asteraceae) AV-044	Scarola selvatica	Leaves	Decoction	Oral	**Med**: diuretic	**1**
					**Cul**: as vegetable in pizzas and soups	**6**
Lathyrus sativus L. (Fabaceae) AV-045	Cicerchie, Chicherche	Seeds		Oral	**Cul**: soups	**4**
Laurus nobilis L. (Lauraceae) AV-046	Alloro, Lauro	Leaves	Liquor	Oral	**Med**: digestive	**3**
			Infusion		**Med**: digestive	**3**
			Decoction (with *Salvia rosmarinus* and *S. officinalis* leaves)		**Med**: flu	**4**
			Decoction		**Med**: calming	**2**
					**Med**: stomach ache	**1**
					**Med**: analgesic	**1**
			Decoction (with *Malus domestica* and *Prunus armeniaca* leaves and *Tilia cordata* flowers		**Med**: sore throat	**2**
			Decoction with *Cynodon dactylon* roots, *Malva sylvestris* leaves and *Matricaria chamomilla* inflorescences		**Med**: bellyache (including dysmenorrhea pains)	**2**
		Fruits	Oil obtained by the fruits	Topical	**Med**: rheumatism	**2**
					**Cul**: to season boiled chestnuts, roast meats, jellied pork, pork liver, sausages	**15**
Lavandula angustifolia L. (Lamiaceae) AV-047	Lavanda	Flowers and Leaves	Infusion	Oral	**Med**: calming	**1**

		Aerial parts			**Dom:** to perfume clothes and domestic spaces	**1**
Linum usitatissimum L. (Linaceae) AV-048	Lino	Seeds	Oil obtained by the seeds	Topical	**Med**: burns	**2**
Logfia gallica (L.) Cosson & Germ. (Asteraceae) AV-049	Bambagia, Vammace	Flower buds	The cotton wool of flower buds	Topical	**Med**: skin disinfectant	**1**
Malus domestica Borkh. (Rosaceae) AV-050	Mele	Leaves	Decoction with laurel and apricot leaves and linden flowers	Oral	**Med**: sore throat	**2**
		Fruits	Boiled slices with vinegar	Topical	**Med**: skin imperfections, cellulitis	**1**
Malva sylvestris L.; *M. neglecta* Wallr. (Malvaceae) AV-051-052	Malva, Mareva	Leaves, Roots	Decoction	Oral	**Med**: toothache	**3**
					**Med**: ovary inflammation	**1**
					**Med**: diuretic	**2**
			Raw	Topical	**Med**: gingivitis	**2**
		Aerial parts	Decoction with *Cynodon dactylon* roots and *Matricaria chamomilla* inflorescences	Oral	**Med**: bellyache	**14**
			Decoction		**Med**: stomach ache, digestive	**10**
					**Med**: flu, cold, cough	**11**
					**Med:** sore throat	**3**
					**Med**: cystitis	**2**
					**Med**: calming	**4**
			Boiled	Inhalation	**Med**: headache	**1**
					**Med**: pimples	**1**
				Topical	**Med**: wound healings	**1**
		Leaves	Three parts of chopped leaves are boiled with four parts of butter until all the watery part has evaporated	Topical	**Cos:** anti-wrinkle	**1**
		Whole plant	Decoction of mallow with bran and cornmeal	Oral	**Vet**: to promote the expulsion of the placenta	**1**
Marrubium vulgare L. (Lamiaceae) AV-053	Marrubio, Marugia, Maruggia	Flowering tops	Infusion	Oral	**Med**: flu	**3**
					**Med**: bellyache	**1**
					**Med**: asthma and cough	**1**
		Whole plant			**Med**: calming	**1**
			Boiled	Topical	**Med**: skin infections	**2**
			Boiled	Topical	**Vet**: skin infections	**3**
Matricaria chamomilla L. (Asteraceae) AV-054	Camomilla, Capommilla	Flowerheads, leaves	Infusion	Oral	**Med**: stomach ache	**12**
					**Med**: bellyache	**19**

					**Med**: calming	**7**
					**Med**: flu	**3**
					**Med**: toothache	**1**
					**Med**: headache	**1**
					**Med**: sore throat	**1**
					**Med**: dysmenorrhea	**4**
			Compresses (also decoction with *Lactuca sativa* leaves)	Topical	**Med**: conjunctivitis	**5**
			Infusion		**Cos:** lighten hair	**1**
Medicago sativa L. (Fabaceae) AV-055		Leaves	Raw	Oral	**Med**: antibacterial	**1**
Mentha pulegium L. (Lamiaceae) AV-056	Poliero, Pulejo, Puliero	Leaves		Oral	**Cul**: for flavoring sauces	**18**
Mentha spicata L.; *M. suaveolens* Ehrh. subsp. *suaevolens* (Lamiaceae) AV-057	Menta	Leaves	Raw leaves chewed	Oral	**Med**: halitosis	**2**
			In ice cold water		**Med**: refreshing	**2**
					**Cul**: for flavoring salads and meat	**8**
Mercurialis annua L. (Euphorbiaceae) AV-058	Mercurella, Mercorella	Leaves	Decoction	Oral	**Med**: intestinal problems, laxative	**2**
					**Vet**: intestinal problems	**1**
Musa spp. (Musaceae)	Banana	Epicarp	Raw	Topical	**Med**: eye swelling	**1**
Ocimum basilicum L. (Lamiaceae) AV-059	Basilico, Vasenicola	Leaves	Raw leaves rubbed on skin	Topical	**Med**: keep mosquitoes away	**1**
Olea europaea L. (Oleaceae) AV-060		Fruits	Oil	Topical	**Med**: earache	**1**
					**Med**: to remove calluses	**1**
					**Med**: burns, wound healing	**2**
		Leaves	Decoction	Oral	**Med**: high blood pressure	**2**
		Fruits	Oil in which river fish were fried	Topical	**Cos:** to regrow hair	**1**
		Fruits	Oil		**Dom:** to make soap	**2**
Onobrychis viciifolia Scop. (Fabaceae) AV-061	Lupinella	Leaves			**Cul**: as vegetable	**1**
Ononis spinosa L. (Fabaceae) AV-062	Ominite, Spina olivella	Roots	Decoction	Oral	**Med**: rheumatism	**1**
Origanum vulgare L. subsp. *viridulum* (Martrin-Donos) Nyman (Lamiaceae) AV-063	Origano, Arechena	Leaves	Decoction	Oral	**Med**: dysentery	**1**
			Decoction	Topical	**Med**: pediculosis	**2**
			Maceration in olive oil	Topical	**Med**: rheumatism	**3**
			Maceration in alcohol		**Med**: toothache	**1**
			Decoction in red wine	Topical	**Med**: stiff neck	**2**

		Stems		Topical	**Med**: in the pierced ears to keep the hole from closing and prevent infections	**1**
		Flowering tops			**Cul**: To flavor poultry, fish, and other dishes as a spice in tomato based sauces or salads	**5**
Panicum miliaceum L. (Poaceae) AV-064	Miglio	Stems			**Dom:** brooms	**7**
		Fruits	Boiled		**Cos:** hair masks	**1**
		Seeds			**Vet**: fodder	**1**
Papaver rhoeas L. (Papaveraceae) AV-065	Papagno rosso, Rosolaccio	Aerial parts, Fruits	Boiled	Oral	**Med**: soporific for children	**11**
		Leaves			**Cul**: as vegetable	**1**
Parietaria judaica L. (Urticaceae) AV-066	Paretaria, Evera è muro, Vetraiola	Leaves	Decoction	Oral	**Med**: abortive	**1**
					**Med**: respiratory system	**2**
					**Med**: hepatomegaly	**3**
					**Med**: jaundice	**1**
					**Med**: kidney stones	**1**
					**Med**: cystitis	**1**
			Cataplasm	Topical	**Med**: wound healing, burns, herpes zoster, rhagades	**6**
			Leaves between the bandages soaked in egg white to block the fractures		**Med**: fractures	**2**
					**Dom**: to clean bottles and clothing	**4**
Petasites hybridus (L.) G.Gaertn., B.Mey. & Scherb. (Asteraceae) AV-067	Lappazzio, Lapazio, Farfaraccio	Leaves	Raw	Topical	**Med**: pimples	**3**
Petroselinum crispum (Mill.) Fuss (Apiaceae) AV-068	Prezzemolo, Pretusino	Leaves	Juice	Oral	**Med**: abortive	**2**
Populus nigra L., *P. alba* L. (Salicaceae) AV-069	Pioppo	Stems			**Dom:** to make flutes	**1**
Portulaca oleracea L. (Portulacaceae) AV-070	Cientofrunnelle, Pucchiacchella	Leaves			**Cul**: salads	**10**
Prunus avium L.; *P. persica* Batch (Rosaceae) AV-071		Leaves, stems	Decoction	Oral	**Med**: headache	**1**
Prunus armeniaca L. (Rosaceae) AV-072	Albicocche	Leaves	Decoction with *Malus domestica* and *Prunus armeniaca* leaves and *Tilia cordata* flowers	Oral	**Med**: sore throat	**2**
Prunus spinosa L. (Rosaceae) AV-073	Prugnolo, Trigne	Fruits	Liquor	Oral	**Med**: flu	**2**
Punica granatum L. (Lythraceae) AV-074	Melograno	Seeds	Syrup	Oral	**Med**: refreshing, invigorating	**3**
		Epicarp	Boiled		**Med**: dysentery	**1**

		Seeds	Raw	Topical	**Med**: gingivitis	**1**
Ricinus communis L. (Euphorbiaceae) AV-075	Ricino	Seeds	Oil	Oral	**Med**: bellyache	**1**
Robinia pseudoacacia L. (Fabaceae) AV-076	Acacia	Flowers			**Cul**: fritters	**1**
Rosa canina L. (Rosaceae) AV-077	Rosa	Fruits	Decoction	Oral	**Med**: vitamin C supplement	**1**
		Leaves	Decoction, liquor	Oral	**Med**: flu	**3**
		Fruits	Raw	Topical	**Med**: gingivitis	**1**
					**Cul**: jam	**1**
		Flowers	Boiled		**Dom**: to dye fabrics	**1**
Rubus ulmifolis Schott (Rosaceae) AV-078	More, Rovi	Fruits	Liquor	Oral	**Med**: digestive	**1**
					**Med**: flu	**1**
		Leaves	Raw	Topical	**Med**: pimples	**3**
		Stems			**Dom**: brooms	**1**
		Fruits			**Cul**: jam	**4**
		Stems	Infusion	Oral	**Vet**: coryza of hens	**1**
Rumex crispus L. (Polygonaceae) AV-079	Lingue di cane	Leaves	Raw	Topical	**Med**: pimples	**2**
Ruta graveolens L.; *R. chalepensis* L. (Rutaceae) AV-080-081	Ruta	Aerial parts	Raw, infusion, liquor	Oral	**Med**: flu, cold, cough	**5**
			Ground with garlic and filtered	Oral	**Med**: stomach ache	**5**
		Leaves	Raw, infusion	Oral, inhalation	**Med**: bellyache	**2**
			Decoction	Oral	**Med**: colitis	**1**
					**Med**: menstrual disorders	**1**
					**Med**: arthritis	**1**
					**Med**: abortive	**1**
			Raw		**Med**: ringworms	**4**
					**Med**: invigorating	**1**
			Boiled	Topical	**Med**: toothache	**4**
			Raw		**Med**: eye swelling, to improve vision	**2**
		Whole plant	Fried in olive oil		**Med**: bruises	**1**
Salix alba L. (Salicaceae) AV-082	Salice	Stems			**Dom:** baskets	**5**
					**Dom**: to tie the vine to the posts	**2**
					**Dom:** to make brooms	**1**
					**Dom**: to make flutes	**2**
Salvia officinalis L. (Lamiaceae) AV-083	Salvia	Leaves	Infusion with *Laurus nobilis* and *Salvia rosmarinus* leaves	Oral	**Med**: flu	**2**
			Infusion		**Med**: asthma	**1**
			Decoction with lemon juice		**Med**: digestive, stomach ache	**1**
			Raw		**Med**: halitosis	**4**

			Decoction	Oral, topical	**Med**: sore throat	**3**
			Boiled	Topical	**Med**: toothache, whiten teeth	**3**
			Infusion (the filtered water is used to rinse the hair after shampooing)		**Cos**: darken hair	**1**
					**Cul**: flavoring, fritters	**7**
Salvia rosmarinus Schleid. (Lamiaceae) AV-084	Rosmarino, Spinaddosso, Spigaddosso	Flowers, Leaves	Infusion with *Laurus nobilis* and *Salvia officinalis* leaves	Oral	**Med**: cold, flu	**3**
		Leaves	Boiled	Inhalation	**Med**: cold	**1**
		Flowers	Decoction with *Laurus nobilis* leaves	Oral	**Med**: digestive	**1**
		Leaves			**Cul**: flavoring for meat roasts and pasta	**9**
Sambucus nigra L. (Caprifoliaceae) AV-085	Sambuco, Saucco, Sauco, Parmiccole	Aerial parts	Liquor	Oral	**Med**: flu	**2**
			Decoction		**Med**: cold, flu	**3**
			Crushed with garlic	Topical	**Med**: burns	**1**
		Leaves	Boiled		**Med**: leg swelling, mastitis	**3**
		Flowers	Boiled		**Med**: rheumatism	**1**
			Infusion		**Med**: stye	**1**
				Oral	**Med**: excessive sweating	**1**
					**Med**: cough, anti inflammatory	**3**
					**Med**: bellyache	**2**
			Boiled	Inhalation	**Med**: cold	**2**
		Bark	Poultices	Topical	**Med**: sprains	**2**
		Flowers			**Cul**: fritters	**1**
		Leaves	Boiled	Topical	**Vet**: mastitis	**2**
				Topical	**Vet**: animal coat infections	**2**
Sambucus ebulus L. (Caprifoliaceae) AV-087	Saucco elleboro	Fruits			**Dom:** ink	**1**
Saponaria officinalis L. (Caryophyllaceae) AV-088	Erba della schiuma	Leaves, flovers and stems		Topical	**Cos:** shampoo, soap	**2**
Scolymus grandiflorus Desf. (Asteraceae) AV-089	Cardone	Leaves, Stems			**Cul**: as vegetable	**2**
Scolymus hispanicus L. subsp. *hispanicus* (Asteraceae) AV-090	Cardoncello	Leaves, Roots			**Cul**: as vegetable	**2**
Sedum cepaea L. (Crassulaceae) AV-091	Borracina	Leaves		Topical	**Med**: wound healing, burns	**7**
Solanum melongena L. (Solanaceae)	Melanzane	Fruits	Raw	Topical	**Med**: hemorrhoids	**1**
Solanum tuberosum L. (Solanaceae)	Patate	Tuber	Raw	Topical	**Med**: burns	**5**
					**Med**: headache	**1**

Sonchus oleraceus L. (Asteraceae) AV-092	Seone	Leaves	Raw	Oral	**Med**: stomach ache	**2**
			Decoction		**Med**: rheumatism	**1**
					**Med**: blood circulation	**1**
		Leaves	Boiled, raw		**Cul**: as vegetable	**12**
Sonchus asper (L.) Hill subsp. *asper* (Asteraceae) AV-093	Seone	Leaves	Boiled		**Med**: diuretic	**1**
Sonchus tenerrimus L. (Asteraceae) AV-094	Seone	Leaves			**Cul**: salads	**1**
Sorbus domestica L. (Rosaceae)	Sorbe, Sorva	Fruits		Oral	**Med**: laxative	**1**
					**Cul**: jam	**1**
Sorghum bicolor (L.) Moench (Poaceae) AV-095	Saggina, Meleche	Stems			**Dom**: brooms	**1**
Taraxacum campylodes G.E.Haglund. (Asteraceae) AV-096	Tarassaco	Aerial parts	Decoction	Oral	**Med**: diuretic	**3**
		Aerial parts			**Cul**: as vegetable	**1**
Thymus pulegioides L., *Th. longicaulis* C. Presl. (Lamiaceae) AV-097	Timo	Leaves			**Cul**: aromatizer	**2**
Tilia cordata Mill., *T. platyphyllos* Scop. (Malvaceae) AV-098	Tiglio	Flowers	Decoction	Oral	**Med**: sore throat, flu, cold	**7**
					**Med**: stomach ache	**2**
					**Med**: calming	**3**
Triticum turgidum L. subsp. *durum* (Desf.) Husn., *Triticum aestivum* L. (Poaceae) AV-099-100	Grano, Vrenna, Caniglia	Fruits	Toasted flour and bran in a stocking and applied to the cheek	Topical	**Med**: toothache	**1**
			Flour mixed with water and salt and applied on the sore part		**Med**: sprains and contusions	**1**
			Toasted bran in a stocking and applied to the breast		**Med**: pneumonia	**1**
			Bran mixed with egg whites		**Med**: sprain	**3**
			Bran mixed with an egg and applied on the legs		**Med**: leg swelling	**1**
		Stems	Hay		**Med**: leeks	**1**
					**Dom**: straws to drink liquids	**1**
Urtica membranacea Poir., *U. dioica* L. (Urticaceae) AV-101	Ortica, Ordiche, Vurdica, Vicci	Roots	Decoction	Topical	**Med**: eczema	**2**
				Oral	**Med**: purify the blood	**1**

		Leaves	Raw		**Med**: digestive	**1**
			Infusion		**Med**: menstrual disorders	**1**
					**Med**: hemorrhoids	**1**
		Whole plant	The painful parts are scourged with the stem of the freshly harvested plant	Topical	**Med**: rheumatism, sciatica	**1**
		Leaves	Infusion	Topical	**Cos**: hair care	**10**
		Leaves		Oral	**Cos**: skin care	**1**
					**Cul**: as vegetable	**10**
			Decoction		**Vet**: cure poultry	**4**
Ulmus minor Mill. (Ulmaceae) AV-102	Olmo, Puleja	Bark	Raw	Topical	**Med**: wound healing	**8**
					**Vet**: wound healing	**2**
Valeriana officinalis L. (Valerianaceae) AV-103	Valeriana	Aerial parts	Decoction	Oral	**Med**: calming	**1**
Vitis vinifera L. (Vitaceae)	Vite	Fruits	Boiled wine	Oral	**Med**: cold, cough	**2**
					**Cul**: vincotto	**2**
		Bark	Raw		**Dom**: smoked	**1**
Zea mays L. (Poaceae) AV-104	Granone, Graulino	Leaves			**Dom**: mattress	**1**
